# Hydrocephalus 2008, 17–20^th ^September, Hannover Germany: a conference report

**DOI:** 10.1186/1743-8454-5-19

**Published:** 2008-12-16

**Authors:** Hazel C Jones, Petra M Klinge

**Affiliations:** 1 Gagle Brook House, Chesterton, Bicester, Oxon, OX26 1UF, UK; 2 Neurosurgical Department, International Neuroscience Institute Hannover, Rudolf-Pichlmayr-Str. 4, 30625 Hannover, Germany

## Abstract

Hydrocephalus 2008 was held 17–20 September in Hannover, Germany, at the invitation of Petra M Klinge (President), co-hosted by Joachim K. Krauss (Vice President), and Madjid Samii (Honorary President). This meeting was a successor to Hydrocephalus 2006 held in Göteborg, Sweden, organised by Past-President, Carsten Wikkelso. The conference began with a general introductory session of six talks including three invited lectures, followed by eighteen parallel sessions. Subjects covered were hydrocephalus signs, symptoms and diagnosis, especially in normal pressure hydrocephalus; cerebrospinal fluid (CSF) physics and dynamics; CSF function and modelling of function; dementia and quality of life, economy, health care and rehabilitation; neuropsychology, cognition and outcome assessment; neuroimaging, functional imaging and non-invasive diagnostics; paediatric and adolescent hydrocephalus; intelligent shunt and valve design (e.g. telemetry, adjustable and antimicrobial shunts); endoscopic third ventriculostomy; technical advances and image-guided surgical approaches in the treatment of hydrocephalus; brain metabolism, biomarkers and biophysics; co-morbidity, classification and aetiology; epidemiology, registries and clinical trials; experimental hydrocephalus; and pharmaceutical modulation of central nervous system function (CNS drug delivery). Each session began with introductory talks from the invited chairpersons followed by six to eight submitted oral presentations. Overall, 136 oral presentations and 18 posters were presented, the abstracts of which were published elsewhere [1]. We present here an account of the introductory session, the invited chairperson's talks and the concluding remarks by Anthony Marmarou.

## Introduction to Hydrocephalus 2008

*Welcome to Hannover, welcome to 'Hydrocephalus 2008'*. Madjid Samii

Madjid Samii as Honorary President, welcomed participants to Hannover for Hydrocephalus 2008, in particular Tony Marmarou, a pioneer and leader in the field for hydrocephalus research for over 30 years, and Peter Black as Guest of Honour and President-Elect of the World Federation of Neurosurgical Societies. He thanked Carsten Wikkelso for organising the previous meeting in 2006, and for having Petra Klinge host Hydrocephalus 2008.

Hydrocephalus is a problem that neurosurgeons face every day and the onset of hydrocephalus with an increase in intracranial pressure is a permanent sequel to neurosurgery. Neurosurgeons need to understand the variable pathophysiology of hydrocephalus and at the same time to search for the best treatment option. Hydrocephalus has many causes, some not fully understood, and neurosurgeons puzzle over why only some patients develop hydrocephalus, the poorly-understood pathology, and why these patients react so differently to treatment. The objective for this meeting was to provide a forum for evaluating experiences, and for solving ongoing problems for patients.

*Introduction to the meeting report on Hydrocephalus 2008*. Petra M. Klinge, President of Hydrocephalus 2008.

After the success of the first multi-/interdisciplinary international conference in Göteborg, this second conference was planned to take place in Hannover, Germany, in September 2008. The format introduced at Hydrocephalus 2006 was adopted in 2008, in that the session chairs presented introductory state-of-the-art lectures, together with recommendations for future directions.

Hydrocephalus 2008 aimed to cover both the traditional (established), as well as the more non-traditional concepts of hydrocephalus research. Traditionally, hydrocephalus has been approached through an understanding of fundamental changes in the brain and the cerebrospinal fluid (CSF) physics and dynamics. Both clinical and experimental research on brain metabolism and chemistry and on aspects of brain function and functional outcome, promise a better insight into the disease. However, these aspects have not been emphasised at scientific conferences and as yet, have not been used in clinical decision making for shunt treatment, for novel treatment options, or outcome assessment of hydrocephalus.

Cognition and the life-time cognitive deficit in hydrocephalus are increasingly recognized as a paramount burden to patients that impact on the results of shunt treatment. As such, clinicians have to understand cognitive domains and cognitive function in hydrocephalus, neuropsychological assessment and aspects of neurorehabilitative treatment. This calls for inter-disciplinary and multidisciplinary cooperation and a disease approach, as for example, in the dementia and co-morbidity of dementia in idiopathic normal pressure hydrocephalus (NPH).

It is now understood that so far, the shunt has not resolved all aspects of the disease, and maintaining favourable long term outcome in patients is constrained by various technical and physiological obstacles. The use of pressure-adjustable valves, novel approaches in shunt and valve design and invasive measures to approach disease mechanisms, are promising. It requires physicians, researchers, engineers, the medical industry and manufacturers to establish mutual exchanges of basic scientific knowledge, experience and scientific achievements. Furthermore, indications for endoscopic third ventriculostomy (ETV) are currently expanding. ETV is increasingly used as the first-choice-treatment instead of shunt treatment in both communicating and non-communicating hydrocephalus because it may provide a physiological restoration of CSF pathways and CSF dynamics.

Universal guidelines in hydrocephalus have been absent through a lack of prospective and standardized trials to support recommendations for standards of care. As a consequence, prospective multicenter trials involving standardized reporting of diagnosis, treatment and outcome in a larger number of patients are evolving around the world. Also, and probably a point of utmost concern, attempts at a better classification scheme have been put forward. Consequently, topics were chosen for this conference based on the above issues, hoping to stimulate new facts and novel interpretations. Novel aspects were sessions dedicated to research in experimental hydrocephalus and to pharmaceutical modulation of the CNS (CNS drug delivery), in order to underline and pursue the translational aspect of hydrocephalus research. For example, recent research findings on neuromodulation in neurodegenerative diseases will eventually translate into an adjunct treatment arm for hydrocephalus in the future.

Accordingly, the conference succeeded in recruiting an inter- and trans-disciplinary team of experts, speakers and chairs, coming from the various professions and groups involved in the treatment and care of patients with both paediatric and adult hydrocephalus and dementia and involved in neurological research [see additional file 1]. The focussed talks from the session chairs, plus the lectures in the opening and closing sessions of the conference, are summarized in this report.

## Introductory session

*Paradigm shifts among a multitude of axes – Hydrocephalus 2008*. Joachim K. Krauss

There have been several meetings worldwide, focussed on hydrocephalus, just to mention the last one, the International Hydrocephalus Workshop 2007, organized by Anthony Marmarou in Rhodes in 2007. When hydrocephalus is discussed, many different aspects of neurosurgery and neurology are included. However, when we look back in history, only 50 years ago hydrocephalus was a historical curiosity. Normal pressure hydrocephalus (NPH) was discovered only 40 years ago. Even now, the whole picture is not yet clear and it is necessary to raise awareness of hydrocephalus. Hydrocephalus may be the most common disorder treated worldwide, it creates a major burden of suffering and there are many humans with shunts all around the world.

Journals and newspapers need to draw attention to the disease. For example, recently the journal *Focus *had a unique article stating 'It was the hydrocephalus' about a patient long suffering from NPH and facing various obstacles before being adequately diagnosed and treated. The patient is now back to his previous life and enjoying his motorbike. It is hoped that by increasing public awareness of the potential for a cure, patients can be treated earlier and more efficiently. According to a survey on practitioners in North America, published by Dr. H. Conn, an emeritus physician from Yale University and suffering from normal pressure hydrocephalus (NPH), one third of the physicians had never heard about this condition. Even among specialists, knowledge is poor regarding the appropriate diagnosis of NPH. A survey in neurosurgical centres in Germany in 1999 showed that most centres obtained CSF tap test volumes anywhere between 0 and 20 ml. Also, there was no consensus for cut-off limits for CSF tap test or for CSF B-wave frequency and amplitude. So there an urgent need to come to an agreement regarding what we think hydrocephalus is and what it is not.

Paradigm shifts have occurred in hydrocephalus, e.g., the concept of longstanding overt ventriculomegaly (LOVA), which gives entirely new perspectives for treatment. Also we understand that NPH is not only a disorder of CSF dynamics, but is also a disorder of the brain. Finally, progress has been made with endoscopic third ventriculostomy (ETV) with more indications and potential and there has been considerable improvement in shunt technology in the past five years. To summarize, in the 60s and 70s we had the period of optimism, in the 80s and 90s there was period of nihilism, and now have to come to the period of realism and we have to see what will be in 10 years from now. There are a lot of 'hidden traces' and 'lost highways' in hydrocephalus, however, both, the more attractive and the less attractive topics are circulating at conferences, and it is hoped that we are able to create a balance between all important relevant topics helping to improve interdisciplinary hydrocephalus research.

*Challenges in hydrocephalus Research*. Carsten Wikkelso

Traditionally, hydrocephalus has been the 'Aschenputtel' or Cinderella, of both neurosurgery and neurology. To overcome this and establish a solid ground for developing the care of patients with hydrocephalus we must concentrate on remaining challenges in hydrocephalus research. First of all we should come to an agreement on definitions for the disorder (hydrocephalus) and the specific causative or idiopathic diseases with hydrocephalus like stenosis of the aqueduct. The treatment of children and young adults with so-called arrested or asymptomatic hydrocephalus is a controversial subject. We need to answer whether these individuals are harmed more by living with an enlarged ventricular system than by being shunted. Past studies have indicated that shunt treatment improved children and young adults with arrested and asymptomatic hydrocephalus.

The most important challenge for the hydrocephalus community is probably to establish a common scale for evaluation of symptoms and signs. To agree on a common scale is a basic requirement for interpretation of symptoms and signs and improvement after surgery reported in different studies. Such a scale should contain continuous variables and high validity, reliability, sensitivity, ability to detect changes, appropriateness, feasibility and interpretability. At the moment we are operating on only 20 to 25% of the patients with idiopathic normal pressure hydrocephalus (iNPH) according to recent studies of prevalence and incidence. Why is that? One reason might be lack of awareness among physicians. Another reason might be that the symptoms often resemble those of other, not yet treatable, brain disorders such as Binswanger's disease, a small vessel disease affecting mainly periventricular white matter. A third reason might be that the available diagnostic tools have limited sensitivity and specificity and poorly predict postoperative outcome, and fourthly the surgery is relatively minor but has the potential for many complications.

Concerning symptoms and signs, we have made improvements and iNPH is no longer just the triad but a syndrome characterised besides gait, mental and bladder symptoms, by interrelated postural dysfunction and other motor impairments. The gait disturbance in iNPH has been further characterised clinically and neurophysiologically as reduced gait velocity, broad based, diminished stride length, reduced foot to floor clearance, increased foot angle and freezing of gait. An important feature requiring further evaluation is characteristic retropulsion, which explains up to one third of gait and balance problems. A number of recent studies on the neuropsychological profile of iNPH have revealed important results. All tested domains that were impaired in iNPH patients, improved after shunt surgery, inversely in relation to the degree of prior impairment. Enhancing the sensitivity, specificity and predictive values of the available diagnostic tools are ongoing studies. The best predictive test is probably the external lumbar drainage test, but even so, up to 20% of patients who would be excluded from shunt surgery by that test, would improve with surgery.

Disturbed CSF dynamics induce metabolic and degenerative changes in the periventricular brain tissue. It is well known that cerebral blood flow (CBF) is reduced globally, and regional reductions have been reported in frontal lobes and hippocampus, thalamus and basal ganglia and periventricularly. Oxygen metabolism is reduced in the basal ganglia and periventricular regions; in most cases above penumbra level with intact auto-regulation. CSF biomarkers have shown interesting changes in iNPH. CSF TNF-α (a pro-inflammatory cytokine involved in apoptosis and toxic to oligodendrocytes) is very high before surgery and normal after surgery indicating that the altered CSF dynamics radically changed the inflammatory state. Neurofilament protein an axonal marker has been reported as increased in several studies, indicating dysfunction or damage to the axons. Sulfatide, a marker of demyelinisation, has been reported increased in patients with Binswanger's disease but normal in NPH patients, potentially making it a good differentiating marker. The water content measured as apparent diffusion coefficient or fractional anisotropy is high in the periventricular tissue in acute hydrocephalus but normal in chronic or NPH patients. Magnetic resonance imaging (MRI) is a promising technique with many methodological opportunities. International co-ordination of protocols might be important for establishing methods for visualising cortical and periventricular diffusion, perfusion, metabolic state and reversibility.

*Idiopathic normal pressure hydrocephalus (iNPH) versus longstanding overt ventriculomegaly in adults (LOVA) – Current Status and Modern Concept of Adult Hydrocephalus*. Shizuo Oi

Twenty five years after Hakim and Adams introduced the term normal pressure hydrocephalus, the unique concept of LOVA was introduced. Although the clinical picture has similarities with NPH, the aetiological and pathophysiological aspects are different. LOVA is an infant-type hydrocephalus with severe ventriculomegaly and asymptomatic except for headache and macrocephaly, but which turns to high pressure symptomatic hydrocephalus in adulthood. In the first series of 16 cases reported, headache was present in 15, but also gait disturbance was seen in 12, dementia in 11 and urinary incontinence in 8 patients. Patients had aqueductal stenosis; hence LOVA is a non-communicating hydrocephalus. New concepts in the definition of adult-type hydrocephalus are needed. The definition of NPH has always created confusion and controversy. The triad (gait, mental and bladder symptoms) has never been specific to iNPH but can occur in any type of adult hydrocephalus. Also, the intracranial pressure (ICP) dynamics are not always within the normal range and iNPH can be confused with high pressure hydrocephalus. In some iNPH patients, CSF dynamic tests and imaging may show that the hydrocephalus is not truly communicating.

Based on the experiences with LOVA, hydrocephalus should be clearly defined from the changes over time and not purely on a one-time ICP dynamic study. Although all types of adult hydrocephalus, both secondary and idiopathic, start with high pressure in the acute phase, they may change in the chronic state, exhibiting a normal range of ICP and normal dynamics, independent of the cause and site of obstruction. In the intermediate-term, or during disease progression, they may have variable baseline pressures with or without wave-like elevations. During this chronological continuum, some patients present with progressive hydrocephalus, and hydrocephalic dementia (HD) is one prominent symptom.

Probably, only a small group of adults with hydrocephalus have true NPH, as defined by normal baseline ICP, clinical symptoms that may or may not exhibit the elements of the triad, and CT/MRI exhibiting ventriculomegaly with obliterated cortical sulci. In a series of hydrocephalic adults, we found 20 LOVA, 13 HD and 3 true NPH patients. Ventriculo-Cisternography is recommended as an important diagnostic element in adult-type hydrocephalus as it identifies the regular CSF pathway and may reliably confirm aqueductal stenosis.

In appreciation of Dandy's pioneering work on classification of hydrocephalus, we must distinguish between obstructive and non-communicating hydrocephalus because obstructions can occur anywhere along the pathway of CSF flow. Probably the only true non-obstructive types of hydrocephalus are venous sinus thrombosis, and choroid plexus papilloma leading to CSF overproduction. Any attempt at a better classification should consider onset, causes, underlying lesions, symptoms, CSF circulation and ICP dynamics, changes over time and post-shunt or ETV response. Certainly, therapeutic specificity should be emphasized since the various responses to shunt treatment or CSF drainage can only be explained by individual variations in the capacity for CSF production and absorption. Some patients with true NPH are only treatable with low-pressure shunts or an extremely low-pressure valve system. As such, shunt treatment response depends on each individual's capacity for CSF production and absorption. LOVA is treated with third ventriculostomy, however, with severe ventriculomegaly and a thinned cortical mantle, it needs a delicate endoscopic technique with freehand manoeuvres to control actions and fine instruments. LOVA is probably the most mysterious observation in hydrocephalus since we still do not understand, why severe but asymptomatic ventriculomegaly in a 14 year old girl with a verbal IQ of 150, can turn into symptomatic hydrocephalus in late life.

*Future of academic neurosurgery and the future of hydrocephalus*. Peter M Black

Neurosurgery is an important discipline in hydrocephalus management, and therefore its future may have important implications for hydrocephalus. This report outlines three important elements of academic neurosurgery–interdisciplinary work, innovation, and international relationships and comments briefly on their relation to the hydrocephalus initiative represented at this meeting.

Most advances in science and clinical care are now being made through interdisciplinary cooperation. Neurologists, neurosurgeons, nurses, physical therapists, basic scientists, engineers, and other medical specialists are all involved in hydrocephalus and their voices should all be heard as we move forward in this field. In the future, the leader who can mobilize and coordinate multidisciplinary groups is most likely to achieve major advances both in clinical and research work in hydrocephalus. Examples of such leadership exist in the 52 year-old Society for Research into Hydrocephalus and Spina Bifida and the newly-formed International Society for Hydrocephalus and CSF Disorders.

Innovation is a second critical component of academic neurosurgery in relation to hydrocephalus. Harvey Cushing's 1925 book *The Third Circulation and its Channels *was an example of the importance of this area in neurosurgery. Lewis Weed in Cushing's laboratory at Harvard made major contributions to this field. His work led to two different approaches to CSF physiology at Harvard that were championed by Edgar Bering involving pulsations, and the steady-state analyses of John Pappenheimer. The innovative work of Holter and others led to reliable valve technology. In the 1980's, Carlos Hakim working in our CSF laboratory developed the concept of a programmable valve which has dramatically changed shunt design. Basic physiology, engineering, imaging, and clinical trials are all important areas for innovation in neurosurgery and especially in hydrocephalus.

Finally, international initiatives are critical in the future of neurosurgery and hydrocephalus. The Society for Research into Hydrocephalus and Spina Bifida and the new International Hydrocephalus Society are groups which exemplify the union of many countries in pursuing a particular problem. The World Federation of Neurosurgical Societies, which represents 110 neurosurgical societies around the world and provides instruments and training to neurosurgeons in developing countries, is another example of globalism in neurosurgery. The problems of hydrocephalus, of deciding whether third ventriculostomy or shunt placement is the best approach, of developing inexpensive shunts, and of assessing our management of hydrocephalus in paediatric and adult life, is a great example of the importance of international initiatives in the present world.

Interdisciplinary efforts, a focus on innovation, and emphasis on international collaboration are three of the most important elements of academic neurosurgery and academic medicine in general. Hydrocephalus and the meetings that bring scientists and clinicians working on it together are prime examples of these elements.

## Hydrocephalus – signs and symptoms

*The gait disorder of normal pressure hydrocephalus*. Henning Stolze

The gait disorder of NPH is typically hypokinetic with a reduced velocity due to short and shuffling steps. It is not correct to describe the gait pattern of NPH as ataxic, since ataxia is a typical symptom of a disordered cerebellum. It has been also described as the plate iron gait, since the feet are not raised high enough during the swing phase and are shuffled over the ground. Gait and balance should always be investigated together, since balance is also severely affected in NPH, patients often having a positive history of falls. The pull test, according to the investigation of Parkinson's disease (PD) patients, is mostly abnormal.

Our quantitative investigations, which compared gait in NPH and PD patients, have shown that the most distinctive parameters for differentiation between these disorders are the balance-related gait parameters. In NPH, the step width is enlarged to a broad-based gait pattern. Also the foot angles are abnormal with the feet rotated outwards on the ground. This shows there is an increased need for stability in NPH. Further, PD patients walk with a reduced arm swing compared to NPH. Here arm swing is often exaggerated, so that NPH patients look as though they are propelling themselves forward. However, it is quite difficult to differentiate NPH patients from the gait pattern in microvascular disease (subcortical arteriosclerotic encephalopathy). Here it is helpful to look for asymmetry due to slight hemiparesis.

*Summary of session on hydrocephalus signs and symptoms*. Carsten Wikkelso, (speaking in place of John Pickard)

The papers in this session showed the interrelation between various elements of the symptoms and signs in chronic hydrocephalus; motor, mental and other physical signs. Only formal and standardized assessment and standardized protocols in conjunction with the diagnostic tests, e.g., before and after TAP-test or drainage, or before and after treatment, allow interpretation and understanding of how and which of the symptoms and signs of improvement after surgery, are related to the disease.

## Modelling and physics of hydrocephalus

*Anatomy and biomechanics in hydrocephalus*. Michael Kiefer

Anatomical aspects can explain most clinical signs of hydrocephalus. These are the predisposed sites for ventricular enlargement (e.g. frontal lobes), the long periventricular course of lower limb pyramidal fibres and midbrain structures. Simple physical laws (LaPlace, Pascal) are fundamental for the understanding of hydrocephalus pathophysiology. Recently, traditional pathophysiology has been challenged because elevated resistance to outflow (R_out_) and trans- or intra-mantle pressure gradients have been shown to be inessential for disease progression. Furthermore, transmantle pressure gradients are also absent in non-communicating hydrocephalus.

Apparently, the starting point of hydrocephalus evolution is reduced arterial and/or cranio-spinal compliance hindering the physiological Windkessel effect of the basal arteries, which increases capillary pressure and reduces cerebral blood flow. Both together reduce cerebrospinal fluid (CSF) absorption and elevate R_out _accordingly. Higher capillary pressure results in pronounced brain pulsations which hit incompressible water (CSF) at the inner and outer surfaces. The consequences of Pascal's law and variations in local parenchymal compliance, due to differences between intracranial and venous pressures (Starling mechanism) result in pronounced periventricular destruction. The outer brain is less affected due to greater damping (compliance). Thus isolated ventricular widening despite free communication between the CSF spaces occurs on the basis of reduced compliance and the Windkessel effect. This also explains the close link between hydrocephalus and cerebrovascular diseases.

*The impact of modelling on the 'Renaissance' of intracranial hydrodynamics: *James P. (Pat) McAllister II

The presentations in this session demonstrate the impressive progress that has been made in the last two years in the area of modelling the hydrodynamic effects of hydrocephalus, especially in the area of intracranial pulsatility. While these advances are shedding new light on the association between intracranial pulsatility and hydrocephalus, the cause and effect relationships still are not known. It is quite likely that abnormal pulsatility may impair cerebrovascular function, and technical advances have made it possible to quantify specific changes in parenchymal capillary pulsatility. Future studies should reveal the cellular nature of these changes, now that a few reports have shown that shear forces can cause endothelial cell dysfunction. Nevertheless, it is important to keep in mind that the hydrocephalic brain is much more than a homogeneous 'sponge', with gliosis, inflammation, and microvessel morphology playing important roles. Sophisticated approaches now allow measurement of the viscoelastic properties of the hydrocephalic brain and these properties will soon be evaluated *in vivo*. Finally, the roles that cellular plasticity and physiological compensation play in progressive hydrocephalus, especially in maturing and aging brains, must be incorporated into our knowledge of the pathophysiology and clinical symptoms of this disorder.

## Neuroimaging

*Neuroimaging in normal pressure hydrocephalus: In search of a diagnostic imaging sign – what did we learn so far? *Mats Tullberg

The history of neuroimaging in normal pressure hydrocephalus (NPH) embraces more than three decades. A number of imaging modalities have been used to study anatomy as well as function. While we are still waiting for a major diagnostic breakthrough, recent studies have above all helped us improve our understanding of NPH pathophysiology. Certain anatomical changes are seen in NPH, such as flattening of cortical sulci, widening of temporal horns, a dilated third ventricle, enlarged Sylvian fissures, focally dilated sulci and an increased flow void signal in the aqueduct. None of these signs have proved reliable as diagnostic markers – their presence merely supports the diagnosis. Vascular changes are frequent in the white matter. Other possible white matter changes are chronic ischemia, neuronal dysfunction, loss of white matter integrity and increased water content. Some imaging studies claim to measure viability of neurons. There is evidence of a potentially reversible metabolic disturbance in NPH, affecting different regions such as frontal, parietal and temporal cortex, hippocampi, white matter, mesencephalon, the basal ganglia and thalamus.

A major problem is the large diversity in NPH neuroimaging. Study aims differ, patient groups are often small and the results often contradictory. A task force should be formed to identify the most important issues to solve and urge imaging centres to perform joint studies, thereby gathering large patient samples, using the same diagnostic criteria and evaluation methods.

*Neuroimaging of hydrocephalus: future directions*. Norman Relkin

Neuroimaging plays an important role in the clinical management of hydrocephalus and will likely contribute even more in the future. Quantitative analysis can be used to extract more information from brain images than simple visual inspection allows. Available techniques permit accurate measurements of brain and ventricular volumes from isotropically acquired magnetic resonance images (MRIs). Serial volumetric measurements may help differentiate ventricular enlargement in hydrocephalus from brain atrophy and more sensitively assess shunt response.

There are many types of structural, functional and molecular images that can now be used to map the microscopic integrity, physiology and biochemistry of the brain in hydrocephalus. Diffusion tensor methods permit non-invasive examination of microscopic tissue properties such as diffusivity, anisotropy and tractography. Phase contrast techniques allow quantification of flow in the vascular and cerebrospinal fluid compartments. Functional and molecular imaging methods are also undergoing explosive development and may prove most useful in the differential diagnosis of conditions that mimic or co-exist with hydrocephalus.

To translate these advances in neuroimaging into clinical practice, normative and disease databases must be created using standardized data acquisition and analysis methods that are optimized for hydrocephalus. This task could be expedited by the creation of an international hydrocephalus neuroimaging consortium that assists in multicenter studies and shares methodology as well as imaging data between sites.

## Aspects of CSF and blood flow dynamics and pathology in hydrocephalus

*Vascular components of CSF dynamics – an update*. Zofia Czosnyka

Starting from mid 1980's, many brain imaging studies were focussed on altered cerebral blood flow and its distribution in patients suffering from normal pressure hydrocephalus (NPH). In experimental studies we find evidence that both hypercapnia and hypotension affect resistance to CSF outflow (R_out_), although not very strongly. Hypercapnia increases R_out _and hypotension decreases R_out_. From clinical practice, we know that intracranial pressure (ICP) B waves which are helpful in the diagnosis of NPH, are probably caused by similar fluctuations in cerebral blood flow detectable by transcranial Doppler (TCD). Waves of the same frequency as ICP B waves can be also seen in cerebral blood oxygenation monitored with near infrared spectroscopy. Similarly, plateau waves of ICP, are provoked by intrinsic increase in cerebral blood volume, due to vasodilatation. In idiopathic intracranial hypertension there is a strong link between ICP and sagittal sinus pressure. A rise in ICP provoked by lumbar infusion produces an equivalent rise in sagittal sinus pressure. This is probably due to pressure on the dural sinuses by rising ICP and results in obstruction of venous blood outflow. Pressure reactivity, calculated from variations between arterial pressure and ICP is correlated positively with R_out_. Surprisingly, the character of this relationship reverses after shunting. There is enough evidence that testing CSF dynamics should be supplemented by testing the cerebrovascular reserve- using for example, non-invasive CO_2 _reactivity or modern brain imaging techniques. In NPH, CBF in white matter decreases as a function of distance from the surface of ventricles. In normal volunteers the distribution of CBF is flat. Autoregulation is less efficient closer to surface of ventricles than further away from ventricles. If cerebrovascular defects are severe, no matter how disturbed the CSF circulation, the shunt is unlikely to help.

In conclusion, CSF dynamics and CBF regulation are strongly coupled. The problem is that in pathophysiology of hydrocephalus we still do not know what is a chicken and what is an egg.

*CSF dynamics in the macro and micro environment*. Martin Schuhmann

The bulk flow concept of CSF dynamics has failed to explain other forms of hydrocephalus than that caused by obstruction of the intraventricular CSF pathways or the ventricular outflow. The hydrodynamic hypothesis for hydrocephalus considers in addition to a pure bulk flow problem, the dynamic pulsatile nature of the CSF movement in concert with the venous and arterial pulsatility and the pulsatile movement of the brain itself. Furthermore, the CSF reabsorptive capacity is considered to be *per se *unlimited. CSF reabsorption most likely occurs together with the hundred fold amounts of arterially filtered extracellular fluid, into the venous side of the capillary bed. The hydrodynamic viewpoint can explain how, apart from the intraventricular interruption of the CSF bulk flow, an obstruction of the intraventricular and extraventricular CSF spaces changes local and global compliance and in consequence, pulsatility patterns, which leads to a hydrocephalic condition. The experimental McAllister model of basal cistern obstruction supports this by creating severe hydrocephalus simply by changing the basal cistern properties. In clinical practice therefore diagnostic methods like high resolution MRI for anatomical evaluation of the CSF spaces, multidirectional CSF flow studies for functional analysis, and computerised ICP analysis of pulsatility and compliance, need to be combined to develop an overall understanding of the underlying pathology in individual patients.

## Assessment of CSF dynamics for diagnosing hydrocephalus

Anders Eklund and Nina Andersson

Shunt insertion explicitly changes the CSF dynamics in patients with hydrocephalus, causing many to improve clinically. However, the relationship between a changed hydrodynamic state and improved clinical performance is not fully known. Therefore, further research in this area is an important challenge for the hydrocephalus research community. This work involves development of better methods for assessment of CSF dynamic parameters as well as studies to test hypotheses on relationships between CSF dynamics and outcome after shunting. The aims are for a better understanding of hydrocephalus pathophysiology and to find new predictive tests.

The model of the CSF dynamic system includes a pressure-independent outflow resistance (R_out_), pressure-dependent total craniospinal compliance and a constant CSF formation rate. The pulsatile properties are further described by the relationship between cardiac-related arterial expansion together with venous, intracranial and spinal compensatory properties.

The most basic CSF dynamic measurement is intracranial pressure (ICP). From this record not only can the mean ICP be determined, but physiological variations such as B-waves, respiratory waves and ICP pulsations can also be analysed. Recently, there has been a number of studies suggesting that a large cardiac pulsatility in the pressure recording is a good predictor for a positive outcome from shunting, and also that the pulse amplitude is reduced by shunting. This indicates that hydrocephalus patients have a decreased compliance, possibly due to a slightly increased ICP. After shunting, ICP is reduced giving a better compensatory ability. This also creates a link between the bulk flow theory and the pulsatility phenomenon. Cardiac-related CSF and blood flow measured with phase contrast MRI are another possibility for assessment of CSF dynamics. Increased CSF pulsatility in the aqueduct is suggested as an indication for idiopathic normal pressure hydrocephalus (iNPH). Another iNPH-related assessment is a shorter arterial venous time delay between blood flow in carotid arteries and sagittal sinus, indicating decreased intracranial compliance.

A powerful method for estimating CSF dynamic parameters is obtained by mathematical expressions calculated from the model, together with active infusion of artificial CSF. CSF infusion patterns can be bolus, constant flow or constant pressure. One challenge with CSF dynamic measurements is that the infusion rates are small in comparison to the often large physiological variations. In some patients these variations can reduce the reliability of the determined R_out _considerably. The precision of each individual R_out _measurement should thus be estimated using all three methods. The infusion test for assessment of CSF dynamics is used clinically for diagnosing and predicting the response to shunting and for assessment of shunt functioning *in-vivo*.

## Aspects of valve and shunt dynamics and pathology in hydrocephalus

*Pathologic considerations in hydrocephalus*. Edward Stopa

During early development, the primitive neural tube undergoes a complex sequence of differentiation into the numerous cell types found in the adult brain and spinal cord. Once completed, the brain is designed to remain in a steady state throughout the human lifespan. During the aging process, however, a number of well-recognized changes begin to occur in the brain. The cerebral ventricles increase in size due to a combination of brain atrophy and a degeneration of brain transport interfaces at the level of the blood-brain (BB) and blood-cerebrospinal fluid (B-CSF) barriers. These transport interfaces are essential for maintaining the steady state of the interstitial milieu provided by the extracellular fluid and CSF that is required for normal neuronal function. Aging effects on the choroid plexus and arachnoid granulations lead to a reduction in CSF production and reabsorption, ultimately resulting in the accumulation of harmful proteins. CSF dynamics are very complex and poorly understood. Recent evidence suggests that there is a communication between the subarachnoid space and the nasal lymphatics, and that this pathway may be important for normal CSF drainage. Another area that remains an enigma is syrinx formation and enlargement in the spinal cord. All these aspects and the entire complexity of the CSF dynamics throughout life stages, have to be taken into consideration when trying to understand and evaluate valve and shunt dynamics and failure of shunt treatment in hydrocephalus.

*Hydrodynamic shunt dysfunction in NPH: known and unknown risks and how to avoid them*. Juan Sahuquillo

Lack of clinical collaboration makes it probable that randomized clinical trials are never conducted in the clinical treatment of normal pressure hydrocephalus (NPH). However, a clear trend towards better outcome has been shown in studies published in the last decade, due in part to the availability of more sophisticated shunt hardware. Because there is no robust evidence proving that any valve is superior, neurosurgeons face important dilemmas in choosing the most adequate shunt. This step is essential for improving outcome and avoiding shunt-patient mismatch, a concept coined in cardiac surgery and easily extrapolated to hydrocephalus. One known consequence of hydrodynamic mismatch is shunt over drainage. However, a lesser known consequence of poor hardware selection is occult shunt dysfunction that causes lack of improvement, or initial improvement and later deterioration. Shunt selection must be based on the fact that both ICP and CSF dynamics are highly heterogeneous in NPH. Patients may have active hydrocephalus (mean ICP above 12 mmHg), compensated hydrocephalus (mean ICP 5–12 mmHg) or even low-pressure hydrocephalus (< 5 mmHg). In patients with active or compensated hydrocephalus, we recommend using low or very-low pressure opening ball-in-cone valves together with gravitational devices (GD). G-valves alone or adjustable valves plus GD are alternative options. However, neurosurgeons must be aware that independent studies show a wide variability in the opening pressure between manufacturers, and even between valves of the same manufacturer. In addition, shunt resistance varies greatly, with some valves not suitable for NPH patients. Neurosurgeons should base their choice on information from both independent studies and from that provided by manufacturers.

## Brain biomarkers and brain metabolism in hydrocephalus

*Biomarkers for (differential) dementia diagnosis*. Peter Paul De Deyn and Sebastian Engelborghs

As CSF reflects brain metabolism, CSF biomarkers can be used for unraveling pathophysiological mechanisms and for diagnostic purposes. To identify neurochemical correlates of neuropsychiatric symptoms, we determined biogenic amines and metabolites in CSF by means of HPLC. In frontotemporal dementia, increased dopaminergic activity and altered serotonergic modulation of dopaminergic neurotransmission were associated with agitated and aggressive behavior.

A promising approach to increase diagnostic accuracy is the use of the CSF biomarkers β-amyloid peptide (Aβ_1–42_), tau-protein (tau) and phosphorylated tau (P-tau_181P_). Assessing Aβ_1–42_, T-tau, and P-tau_181P _in CSF from 200 autopsy-confirmed dementia and control subjects, new models have been developed, enabling the use of the different biomarkers in optimal algorithms defined by the clinical need. The models showed promising sensitivity, specificity and diagnostic accuracy levels systematically exceeding 80%. Biomarker-based diagnostic models can as well be applied in diagnostically doubtful cases. Moreover, these biomarkers have a predictive value for conversion to dementia in mild cognitive impairment. In the light of future biomarker applications such as monitoring of disease progression, the concept of *in vivo *surrogate biomarkers should be further explored in order to understand the relationship between circulating biomarkers and pathological mechanisms in the brain.

*CSF biomarkers for chronic adult hydrocephalus: advances and challenges*. Conrad E. Johanson

CSF signal analysis holds considerable promise for more precise diagnosis and management of neurodegeneration. Source, sensitivity and staging are key factors in clinically advancing CSF biomarker utility. Brain, choroid plexus and plasma need to be distinguished as potential sources of CSF proteins and peptides, including amyloid-beta fragments. Augmented permeability (in advanced degeneration) of both the blood-CSF and blood-brain barriers affect marker concentration in CSF. The use of 2D chromatography corrects for the 'swamping' effect of plasma proteins in CSF samples, thereby improving chances of detecting low (but potentially significant) levels of novel biomarkers secreted by choroid plexus and glia (homeostatic response involving immune and stabilizing factors) or catabolites released by disintegrating neurons in disease-affected regions. Combination or ratio analyses of amyloid/tau derivatives in CSF have become increasingly useful as specific indicators of neurodegenerative stage. Longitudinal studies of the CSF composition during progressive aging will promote better interpretation of NPH stages and improve differential diagnosis with CSF biochemistry in Alzheimer's (AD) *versus *other types of dementia. Comprehensive banking of simultaneously procured CSF and plasma samples for aging/NPH/AD, in conjunction with refinements for brain/CSF image biomarking, should provide the requisite information for delineating neurodegeneration and enhancing the CNS response to various therapeutic modalities.

## Classification, etiology and co-morbidity of hydrocephalus

*Toward a definition and classification of hydrocephalus: Why it is important*. Harold L. Rekate 

There is no agreement as to the definition of hydrocephalus, the subject of this entire conference. The currently accepted classification of hydrocephalus was proposed in 1919. Understanding of the condition and development of tools to study the problem have exploded, especially since the development of contemporary neuroimaging. It is essential to develop a consensus on these issues in light of these new discoveries, in order that researchers in diverse fields may develop understanding of the implications and importance of the research efforts of others. A new classification would provide structure to plan and assess ongoing and planned research.

Proposed definition: Hydrocephalus is an active distension of the ventricular system of the brain from the point of CSF production within those ventricles to the point of absorption into the systemic circulation. Proposed classification as modified by the discussion:

A. Hydrocephalus secondary to points of obstruction

1. Foramen of Monro

2. Aqueduct of Sylvius

3. Outlet foramina of the fourth ventricle

4. Obstruction of the basal cisterns

5. Obstruction of distal CSF absorption

6. Intracranial venous hypertension

B. Hydrocephalus without obstruction

1. Choroid Plexus Papilloma

2. Are there any other examples?

The new International Society for Hydrocephalus and CSF Disorders launched at Hydrocephalus 2008 should commit itself to the development of a consensus on these issues.

## Experimental hydrocephalus

*Experimental hydrocephalus: animal models past and present*. Hazel C. Jones

Treatment for hydrocephalus is usually based on some type of surgical intervention. Advances in shunt technology, in imaging and in monitoring techniques, have brought improvements in patient management. Animal models for hydrocephalus have and will continue to have a role in this progress. Specifically, the experimental conditions can be varied to suit the objectives and can be used to test hypotheses. Brain and CSF pathology can be studied before and after shunt treatment.

Four categories of animal model are: CSF obstruction (any age), induction with teratogens (fetal or neonatal), viral infections (neonatal), and genetic (any age, usually fetal or neonatal). Ventricular dilatation is variable depending on method but is generally more severe in young animals. Most models with shunt treatment show partial recovery but some changes are irreversible, particularly in the periventricular white matter.

The numerous teratogenic chemicals that disrupt brain formation, demonstrate that the developing brain is highly sensitive to adverse influences. In these circumstances dilated ventricles can occur without CSF obstruction. Genetic hydrocephalus can arise naturally or be caused by genetic manipulation. Improved knowledge of the genes involved in hydrocephalus is providing information that can be used to investigate unexplained fetal hydrocephalus in humans. In summary, animal models are essential to improve understanding of human hydrocephalus.

*Correspondence between animal models and pathology in human hydrocephalus* Marc Del Bigio. 

Correlations between human disease states and experimental animal models are possible. However, one must always remember the caveats. The basic properties of individual brain cells and their components are similar across mammalian species, but subtle differences exist with respect to molecular and genetic composition. At the organ level, the major components and connections are similar but the ratios differ; non-gyrencephalic animals have less frontal lobe volume and far less white matter. The degree of brain maturity at birth differs widely across mammalian species, and smaller animals mature faster. For hydrocephalus and other neurological disorders one can and should compare behaviour (motor and cognitive), imaging, CSF composition, tissue (histology and biochemistry), and response to drug therapy. Many models of hydrocephalus are available. The initiating factors reflect on human states with varying degrees of fidelity. For example, the blood injection model has strong similarity to post hemorrhagic hydrocephalus. The kaolin, fibroblast growth factor, and transforming growth factor models mimic to some extent the meningeal inflammation and/or fibrosis that occurs after meningitis or hemorrhage (including trauma). Silicone oil, cyanoacrylate glues, and other synthetic polymers effectively obstruct CSF flow in a totally artificial manner. The genetic models (H-Tx rat, L1CAM mouse, ciliary dyskinesia, and others) are useful for various aspects of hereditary and fetal onset hydrocephalus. As in humans, the animal models of hydrocephalus develop head enlargement, motor and cognitive changes, periventricular white matter damage, and they respond to shunting. One can therefore conclude that the animal models of hydrocephalus mimic human pathology to a high degree. However, the experimenter must know the limitations of the model as well as details of the comparative development and neurobiology.

## Treatment of hydrocephalus and management of complications

*Important aspects of complications in shunt surgery*. Bertil Romner

Shunt surgery, is the most common treatment for both paediatric and adult normal pressure hydrocephalus (NPH). The most frequent complications of shunt surgery are: over- and under drainage and infections. Over drainage presents as symptomatic subdural hygromas, haematomas and slit ventricles. Under drainage is either related to obstruction, disconnection, malpositioning, or migration of the shunt system or to a valve with, or set at, excessively high opening pressure, leading to functional under drainage. Less frequent complications related to the surgical procedure are intracranial bleeding or pneumatocephalus, and in the case of adjustable valves, the likelihood for accidental readjustment. The type and rates of complications differ for paediatric and adult hydrocephalus. During the first year after shunt surgery in paediatric hydrocephalus, 10–20% have a shunt infection and about 17% have shunt malfunction. In adult hydrocephalus, the complication rates for surgery are: infection 5–10%, mechanical malfunction 10–30%, and subdural haematoma, 10–15%. In NPH, these complications are rated severe in 20% of patients. Less frequent, but important complications are epilepsy 1–7%, and hearing or visual failure each 2%.

Shunts with adjustable valves enable the functioning pressure to be modified *in situ *and allow non-invasive management of complications such as over drainage and slit ventricles, and under drainage. Adjustments are not always associated with improvement and the management of non-responders needs to be handled more rigorously: patients without clinical improvement in the presence of unchanged ventricular width in spite of several valve adjustments, need a shunt function test followed by a shunt revision if the result is equivocal or positive. To date, these are either radio- nuclide studies or lumbar infusion tests. The lumbar constant rate infusion test allows assessment of the actual valve opening pressure and comparison with preoperative measures: a decrease in resistance to outflow indicates improved dynamic status and a working shunt.

One problem with adjustable valves is that they can become re-set accidentally with magnetic fields, as in MRI, cell phones, headphones and home magnets. For the Hakim-Medos valve, 140 patients undergoing MRI resulted in a valve setting change in 35 (25%). Questions still to be resolved are whether the following situations affect revision rate in individual patients: using a uni-shunt (to avoid disconnections), endoscopic or navigated placement of ventricular catheters, peritoneal versus atrial placement of the distal catheter, the site of the ventricular catheter (occipital *versus *frontal) and the use of adjustable valves.

*Infection as a major complication in shunting and external drainage: *Roger Bayston

Among the complications of cerebrospinal fluid shunting and intracranial pressure management by external ventricular drainage (EVD), infection is one of the most serious. The incidence varies, both in the literature and in clinical experience. Figures cited for shunting are around 5% for adults but three to four times higher in infants less than six months old. The infection rate for EVD is more difficult to determine because of the different case definitions for diagnosis. However, in most centres it is at least 10% when the diagnosis includes ventriculitis. The clinical consequences of ventriculitis include deterioration in cognition, which can result in decline in quality of life so that the patient becomes ineducable, unemployable and profoundly dependent. In ventriculoperitoneal shunts, infection often causes obstruction of the distal catheter and in some cases loss of absorptive capacity in the peritoneal cavity, requiring alternative routing of the shunt. In both shunting and EVD most infections are caused by staphylococci, but gram negative bacteria are also important, especially in EVD, and for the patient on the intensive care unit multi – resistant *Acinetobacter baumanni *is a problem. *Propionibacterium acnes *is an under-detected cause of shunt infection. Despite many studies of varying scientific validity, there is still no clear evidence that perioperative prophylactic antibiotics reduce the infection rate, and in EVD they have been found to increase the proportion of resistant gram negative infections. Antimicrobial catheters have shown benefit in both shunting and EVD, though the role of silver-processed catheters remains undetermined. Treatment of infection should include catheter removal, preferably with intraventricular antibiotics, although linezolid has been found to give high CSF levels after oral administration, and promises to be useful for staphylococcal infection. For *Acinetobacter *EVD infections, intraventricular colistin is safer then intravenous. Risk of infection can be reduced by shorter pre-operative hospital stay, less use of antibiotics with, where possible, shorter courses, locally targeted antibiotics (intraventricular route, antimicrobial catheters), and enhanced state spending on health care.

## Endoscopic third ventriculostomy and management of hydrocephalus

*Endoscopic third ventriculostomy: evolution of indications and technique* Michelangelo Gangemi.

Endoscopic third ventriculostomy (ETV) is a surgical procedure that allows the CSF to flow directly from the third ventricle to the basal cisterns and subarachnoid spaces, thus by-passing the aqueduct and the CSF pathways of the posterior fossa; its indications and the interpretation of its significance in different forms of hydrocephalus have changed since the introduction of this technique into neurosurgical practice over the last twenty years.

While earlier understanding considered ETV as a simple internal shunt which created a CSF diversion requiring patent subarachnoid spaces and adequate resorption to ensure benefit to the patient, recent successful applications in forms of so-called non-obstructive hydrocephalus (i.e. post- infection and hemorrhage), demand new interpretations for the mechanisms by which this procedure works. Restoration of brain pulsatility and physiological CSF dynamics may play an important role in the positive effects of ETV, other than just CSF diversion. Nowadays, despite great technological improvements in the construction of endoscopic devices and a well-established surgical technique, many points still remain controversial and debated, like the usefulness of ETV in other pathological entities such as 'normal pressure' hydrocephalus in old age and hydrocephalus of newborns.

*Endoscopic third ventriculostomy: outcome, controversies and future perspectives*. Joachim K. Krauss

Endoscopic third ventriculostomy (ETV) has been established as a safe treatment for obstructive hydrocephalus in selected patients, with fewer overall complications than shunt insertion. The most common problems for ETV are poor intraoperative vision because of technique (blood leaks), CSF leaks, subdural fluid collection, ventriculitis or meningitis. Some patients fail to improve and a range of studies has demonstrated variable success rates of 50–90%. In addition, there are many serious, but less common complications such as herniation, arrhythmia, injury to brain structures especially important structures in the third ventricle, haemorrhage, ischaemic stroke, and infections. Of upmost concern is sudden death, which may occur in acute as well as in the later follow-up stages, e.g. late death after sudden closure of stoma after successful procedure.

Repeated ETV procedures have higher complication rates than the first intervention (55.5% *versus *10%). Unresolved controversies are the use of ETV in infants <2 years old, in NPH patients, and in previously shunted patients with obstructive hydrocephalus. In infants less than 2 years, the success rate has been low, although recently a small study reported 57% success. In NPH a 69% improvement rate has been reported. Clearly success is dependent on the presence of a patent subarachnoid space. Previously shunted patients have been treated with ETV after shunt malfunction, and a proportion (38–84%) become shunt free as a result, with variable complication rates. Complications are more frequent than in patients with newly diagnosed hydrocephalus, according to a retrospective review of 131 shunted and un-shunted patients from various age groups and with various etiologies treated with ETV. Shunt-related over drainage can be successfully treated with ETV followed by removal of the shunt. Nowadays, the long-held dogma 'once a shunt – always a shunt' is definitely no longer valid.

## Outcome in hydrocephalus and CSF Disorders

*Outcome in hydrocephalus and CSF disorders*. Mark Luciano

The chairs' lectures introduced the problems of outcome analysis in hydrocephalus. Domenico D'Avello from Padova discussed data collection stressing the importance of multi- institutional collaborative registries, such as developed in Italy, to gather more accurate outcome data. The subjective nature of chronic hydrocephalus symptoms and the need to use quantitative measures, restrained interpretation and controls was stressed by Mark Luciano who called for a randomized placebo-controlled crossover NPH outcome study to better evaluate subjective response.

In this session, four subsequent speakers discussed the prediction of outcome in NPH. C. Sprung from Berlin compared outcome with an adjustable system to their own fixed pressure historical controls and described fewer revisions caused by drainage issues. J. Lemcke, from Berlin proposed that a gradual reduction of CSF opening pressure over the first six months of treatment may reduce over drainage symptoms. M. Schuhmann related trial CSF drainage outcome with measurements of CSF reserve capacity, pressure-volume index and elasticity. In addition, they showed the greatest improvement at the one month time point after shunting. On the other hand, N. Chautouras from Greece reported a 95% improvement rate using only imaging and an ICP measurement for NPH patient selection. A serious complication, Dorsal Midbrain Syndrome, was described by M. Kiefer resulting from over-shunting in obstructive hydrocephalus causing possible midbrain injury. This syndrome requires both surgical procedures, to reduce drainage, and symptomatic medical treatment. V.M. Gerganov reviewed hydrocephalus associated with vestibular schwannomas, describing a correlation between the degree of hydrocephalus and size of tumor. However, predicting the need for direct treatment of the hydrocephalus was difficult.

Other CSF disorders, benign intracranial hypertension (BIH) and cerebral cysts were discussed by A. Tarnaris and T. Keinert, respectively. BIH remains a difficult disorder to treat with a 72% and 48% reduction in headaches and visual disturbance, respectively and a 34% shunt revision rate. T. Keinart, from Heidelberg described a valveless cyst ventricular shunt for the treatment of cerebral arachnoid cysts in a series of 6 patients. A sense of the complexity of outcomes in CSF disorders and difficulties in generalizing from small series without controls, pervaded and enlightened the session's discussion.

*The Italian multicenter web-based iNPH study. Analysis of shunt-related complications*. Domenico d'Avella (on behalf of the Italian NPH group of the SINch, Padova, Italy)

A multicenter prospective controlled study was performed for the diagnosis, treatment, and analysis of long-term outcomes in normal pressure hydrocephalus (NPH). The aim of this report was to describe the rate of complications, morbidity, and mortality associated with shunts at the time of the operation and after the surgical procedure.

A total of 143 patients were enrolled, of whom 140 completed their follow-up. Patients were implanted a Codman Hakim programmable shunt valve. Enrolment was based on the criteria of the Italian Study Group of NPH. Median follow-up for the study was 24 months. Perioperative mortality rate was 2.1%, due to pulmonary embolism. Total complication rate was 10.7%, requiring additional surgeries in 7.1%. Total intra-operative complications were 2.8%, and late infection-related shunt malfunctions occurred in 5.7%. We recorded 2.1% of subdural fluid collections, being surgically evacuated in one case and treated with valve pressure readjustment in two cases. Valve opening pressure re-adjustments were necessary in 35% of patients. The increased knowledge of the topic, the modern standard of care of patients, the application of rigid inclusion criteria for selecting appropriate shunt candidates, and the availability of programmable valves, make shunt surgery in NPH patient a safer procedure, as compared to previous data in the literature.

## Technical advances in surgical management of hydrocephalus

*Finding the right treatment*. Magnus Tisell

Technical advances include any innovation which improves the management of the hydrocephalus patient and ideally also the outcome. We could either improve the shunts or make the patients shunt-independent. Factors that can improve shunt surgery are changes valve design, antisiphoning devices, different shunt materials and different design and position of catheters and tubes. The most obvious strategy for shunt-independency is removing an underlying cause such as an obstructive intracranial lesion. An unnecessary shunt should be avoided by improving the criteria for surgery. The most common endoscopic procedures for achieving shunt-independency are ventriculocisternomy and fenestration of cysts. In the future there will probably be effective drug therapies. Many inventions are focused on reducing complications and side effects. Equally important is functional status after optimal treatment. Any new treatment has to be tested against current standards. The placebo effect is well known, but there are also the negative nocebo effects. Long term effects are by definition unknown when a new device is introduced and are particularly important in life-long diseases such as hydrocephalus. In summary, what we look for are long lasting inventions with clear indication(s), few complications, few side effects, and optimal functional outcome. To achieve this, we need excellent clinical science.

*Validation of technical advances in the treatment of hydrocephalus*. Gerald D. Silverberg

New shunt devices and techniques are reported regularly. The benchmark for evaluating clinical devices and techniques is the prospective, randomized double-blinded, placebo-controlled trial. There are pitfalls inherent in designing a well-controlled trial, as exemplified in our trial of low-flow CSF shunting in Alzheimer's disease (AD). We compared a functioning shunt group to an occluded shunt group using a novel, low-flow V-P shunt system. We chose two FDA-approved end-points: the Mattis Dementia Rating Scale (MDRS), and the Global Deterioration Scale (GDS). The generalized estimating equation (GEE) compared the two randomized groups. The study was halted when the GDS did not change over nine months in either group. Several retrospective flaws were identified: 1) the GDS was too insensitive. A secondary activities of daily living measure (ADCS-ADL) showed a benefit, 2) there were too many moderate-severe AD subjects (>30%). We intended to treat only early AD, 3) the shunts used did not perform reliably *in situ*, and 4) the statistical method used was ill-suited to the wide variation in responses in the test group. In two *post hoc *analyses using a linear mixed effects statistical model, the MDRS and the ADCS-ADL as end-points, there was significant benefit of active shunting in early AD, (MDRS p = 0.028, ADCS-ADL p = 0.021)

## Epidemiology, trials and registries

*The Swedish longitudinal adult hydrocephalus surgery registry: *Kristina Cesarini

In 2004 all six University Hospitals in Sweden started a quality registry that provides online data access of the unit's own hydrocephalus figures and the national figures for comparison. All adults = 18 years that underwent first time surgery (shunt and 3^rd ^ventriculostomy) for hydrocephalus are included. The web-based registry is connected online to the national population register that is automatically updated for geographical data and deaths. Hydrocephalus is classified as communicating/non communicating, idiopathic/secondary. Basic demographic data, baseline diagnostic work-up, coexisting diseases and neurological function are registered with validated scales according to a standardised protocol. Main surgical and technical details, complications, reoperations and shunt adjustments are also registered. Follow up function is evaluated at 3 and 12 months postoperatively. Up to September 2008, 1018 patients, 50% females, age 18–88) were registered.

Eighty-two percent had communicating hydrocephalus, of which 42% were idiopathic and 32% after SAH. Sixty-two percent of the obstructive hydrocephalus was secondary to tumour. Adjustable valves were used in 79% of all shunt procedures, third ventriculostomy was performed in 66% of all obstructive hydrocephalus. Three hundred and thirty valve adjustments were done and 201 patients had 269 complications, 92% occurring during the first year after surgery. CNS infections were found in 4%, incorrect shunt placement/displacement in 5%, 4% had CNS/shunt infections and 4% experienced obstruction/disconnection. Improvement after surgery persisted through the follow up year. Twenty percent had repeat surgery due to complications. Serious complications were rare and postoperative haematomas were <0.5%. Although delay in registration is still a problem, we have now a national collaboration and common measurements to evaluate the quality and performance after hydrocephalus surgery.

*Trials, registries and audits – which is best? *Hugh K. Richards

All clinical investigations designed to evaluate novel treatments are potentially subject to bias, and it is important that investigators are aware of both potential sources of error and key principles of study design, in order to reduce them. The two most important sources of bias are selection bias and observer bias which may be substantially reduced by randomization and blinding respectively. A randomized, double-blind clinical trial is regarded as providing the most valued data. However, in the field of hydrocephalus research, where the subject of the trial is a procedure or a device, blinding is rarely achievable.

Audits are generally neither randomized nor blind and may be subject to study management bias. Treatment groups must be handled equally with regard to all the study procedures. This is extremely difficult when cases and controls are studied at different times. However, audit data is often the first data available and may trigger further trials.

Registries are not blind and may be subject to management bias, but because the dataset under investigation may be large, (The UK Shunt Registry currently holds data on over 40,000 procedures) some degree of randomization may be achieved by performing matched-pair studies where controls are randomly selected.

One problem with all investigations is the number of patients required to achieve significance. A sufficiently powered study evaluating a comparatively rare event (such as shunt infection in adults) requires the recruitment of as many as 2,000 patients. This number is reduced in patients at greater risk but will generally require several hundred patients. A study involving shunt revision for causes other than infection will also require long term follow-up. Because of the lack of perfect investigations in hydrocephalus, investigators should be aware of sources of bias and design protocols to minimize them. Furthermore the requirements of science must always be balanced with ethical considerations. Finally, reviewers must be able to evaluate submitted evidence, and not allow authors to make conclusions that are not justified by the data.

## Pharmaceutical modulation of the CNS (CNS drug delivery)

*Interstitial delivery for biological agents in brain tumour therapy*. Peter M. Black and Rona S. Carroll

Interstitial methods for delivering drugs directly to the brain for tumor therapy include cannulas attached to minipumps, encapsulated cells, and microparticles. With the osmotic minipump, a drug solution is infused directly into the brain via a cannula attached to a subcutaneous reservoir, using osmotic pressure to provide a constant delivery rate. This system has been tested in mice with implanted gliomas. Infusion of murine or human endostatin results in reduced blood vessel density, reduced proliferation and increased apoptosis. Cell encapsulation uses implanted genetically engineered cells that secrete a therapeutic protein and are encapsulated in an immunoisolating material (alginate and poly L-lysine). The advantages of this method are that there is minimally invasive surgery, the cells constitutively produce protein and the capsules are biocompatible. Disadvantages are that long term cell viability may be poor and the rate of output may be erratic. Polymeric microspheres are prepared from a polymer and emulsifier with therapeutic compounds trapped inside. They release the drug continuously over time. Microspheres containing the platelet derived growth factor (PDGF) inhibitor, Gleevec, when implanted into mice with gliomas significantly reduce the size of the tumour and increase apoptosis. It is concluded that local delivery to the brain increases the efficacy of therapeutic agents.

*Drug delivery to the brain via the CSF system – perspectives and limitations*. Thomas Brinker

Recent understanding of the molecular pathways in both acute and chronic brain disease has raised the need for efficient drug delivery to the brain, to enable direct interventions in pathological cascades. Different routes have been used: intravascular or local intracerebral application. The intravascular route is limited by the blood brain barrier (BBB), particularly for non-lipophilic substances, but also for most lipophilic agents larger than 500–600 Da. Since modern potential therapeutic agents are either small oligonucleotides and peptides or more complex lipo-proteins (antibodies), penetration through the BBB is essentially nil. Therefore, methods for local intracerebral drug delivery have been pursued in the recent past. Using substances injected or infused into the CSF space, it was demonstrated that the entire brain tissue may be pharmacologically targeted. However, despite the potential for continuous delivery, the effects can be suboptimal because of non-selective receptor activation, and subtherapeutic concentrations because of enzymatic degradation, ependymal receptor binding and/or rapid CSF clearance. The use of genetically engineered cells, which continuously release substances, i.e. neuropeptides, has been investigated recently in the clinical and experimental setting. Here, genetically engineered human mesenchymal stem cells (hMSCs) provide a promising vehicle, as they are non-embryonic, non-tumorigenic, and non-proliferative. Furthermore, encapsulation of those cells in alginate, a biocompatible biopolymer, allows 'immunoisolation' preventing allogenic host-versus-graft reactions, and also allows osmotic passage of nutrients and therapeutic agents. We locally implanted genetically engineered alginate encapsulated hMSCs releasing the neuroprotective peptide Glucagon-like peptide-1 (GLP-1) in experimental traumatic brain injury, and were able to demonstrate significant reduction of hippocampal neuronal loss in the stem-cell treated animals. When it comes to the human application, retrievability of cells is an important safety issue, and we have developed a prototype device for intrathecal implantation of those cells, which is now used in a phase I/II trial in patients with hemorrhagic stroke. It is thereby hoped to improve encapsulated cell biodelivery, also called *ex-vivo *gene therapy, to exploit the CSF system for pharmacological manipulation of the nervous system.

## Cognition in hydrocephalus and related dementias

*The neuropsychology of normal pressure hydrocephalus, established facts and remaining problems*. Per Hellström

Memory impairment, and cognitive deterioration, albeit imprecise and far from synonymous, point to the fact that patients with NPH, in general, undergo mental changes. The cognitive deficits and behavioural manifestations of NPH are often said to indicate or, at least resemble, frontal dysfunction. However, a search of the literature from the mid seventies until the present, gives a picture of more widely distributed pathological changes. Affected neuropsychological and cognitive functions include: abstract thought, apprehension, attention, numeracy, concentration, dexterity, executive function, higher integrative functions, orientation, manipulo-spatial, motor perception, pragmatic language, psychomotor speed, short term memory, verbal language, visual construction and perception, working memory, and writing skills. Reported numerous behavioural and psychiatric changes include: aggression, altered consciousness, apathy, aspontaneity, awakening fluctuations, bradyphrenia, depression, disturbed impulse control, easily fatigued, emotional disturbance, fantastic confabulation, apraxia, impaired wakefulness, indifference, inertness, bipolar disorder, negative frontal syndrome, obsessive compulsive disorder, obtunding, perseveration, positive frontal syndrome, psychosis, and reduced alertness.

Acknowledging the fact that NPH can affect most neuropsychological areas, investigators now need to agree on the selection of a few instruments, capable of reflecting some of the most conspicuous changes. These instruments will have to be proven to be reliable (resisting practice effects), validated (ideally, strongly associated with physiological measures), sensitive (discriminative) and able to detect changes following treatment.

*What can we learn from cognitive neuroscience for hydrocephalus diagnosis? *Thomas Münte

Cognitive neuroscience has a classical tradition based on localisation, the isolation of single cognitive processes, and the technique of minimal contrasts. However, the clinical neurologist needs to know the functional changes in the whole brain network. There are two possibilities for the whole brain approach: functional magnetic resonance imaging (fMRI) that has been used for multiple sclerosis patients, and independent component analysis (ICA)-based definition of resting state networks used in amyotrophic lateral sclerosis (ALS) and dystonia patients.

A method that could be applied to normal pressure hydrocephalus is fMRI plus global cognitive stimulation (GLOSTIM). For this test, a task is performed that taxes many cognitive processes and is combined with fMRI imaging. This gives a picture of diffuse global activation that could be used as an index of destruction and compensation.

The ICA-based method assumes that brain areas are connected to functional networks with neurophysiological activity. The activity is reflected in the fMRI blood oxygen level dependent (BOLD) signal which can be measured during task execution and at rest. ICA analysis is used use to extract the activity due to spatially and temporally independent networks from the spontaneous fluctuations in the BOLD response. The advantages of this method for use in hydrocephalus clinical research are that it is easy to perform in most MR scanners with a run time of 6 min on scanners of 1.5 T or more, does not require any specific equipment, it is not task biased, shows sensitivity to change and has shown a high inter-individual stability.

## Quality of life, social impact, health care and rehabilitation

*Quality of Life: What does it mean to patients and families? *Dory Kranz

From my perspective as a patient advocate, not a scientist, I see that quality of life is highly important to people with hydrocephalus and means essentially the same to them as to any human. In a survey of Hydrocephalus Association community members, overall quality of life was rated # 1 in importance by almost a 20% margin. Quality-of-life markers like education, employment and relationships were three of the next five priorities – ranked in importance between the critical medical issues of shunt malfunction and shunt infection. Dr. Abraham Maslow's hierarchy of needs provides a framework in which people seek successively higher level needs as the more basic needs are met. The advent of shunting and extraventricular drain as treatments, resolved – for the most part – the most basic physiological needs and allowed people with hydrocephalus to stay alive. Research focus then moved to the next level of needs which includes health safety. When individuals with hydrocephalus are not in a life-threatening emergency due to shunt malfunction, infection or some other complication, they seek the next higher level needs of love/belonging (friendship and love relationships), esteem (education and employment) and self actualization. Traditionally, little time has been spent looking at these higher level outcomes scientifically. Some exciting research is beginning to address quality of life: paediatric outcomes research and questionnaire by Abhaya Kulkarni and colleagues at the University of Toronto; cognitive outcome research by David Frim and Maureen Lacy at the University of Chicago; and work by Nalin Gupta and associates at UCSF, to understand long term outcomes for adults treated in childhood.

*Quality of Life: What does it mean? How do we measure it? How can it influence clinical care and research? *Michael A. Williams

The outcomes usually measured in adult or paediatric hydrocephalus (e.g. gait, balance, neuropsychological function, continence, headache) are scientific outcomes and not quality of life outcomes. Health-related quality of life is defined as 'the degree to which persons perceive themselves able to function physically, emotionally, and socially'. Health Status is 'the degree to which a person is able to function physically, emotionally, socially with or without aid from the health care system'. The best body of work in health status measurement in hydrocephalus is by Abhaya V. Kulkarni at the Toronto Hospital for Sick Children. The 51-item Hydrocephalus Outcome Questionnaire (HOQ), administered to parents of children with hydrocephalus, has been carefully designed and validated, and provides an overall score plus sub scores for physical health, social-emotional health, and cognitive health. Variables resulting in lower HOQ scores include seizures and multiple shunt revisions. The mean utility score (a method that allows meaningful comparison between different health status measures) with the HOQ is 0.77, which compares to 0.87 for teenagers born with extremely low birth weight, or 0.85 for paediatric survivors of Hodgkin disease. Quality of life instruments allow hydrocephalus to be compared with other disorders, and can be paired with health expenditure information for cost utility analysis.

## Paediatric hydrocephalus and adolescence

*Challenges in understanding and dealing with paediatric hydrocephalus*. Marianne Juhler

Paediatric hydrocephalus has been categorised as either obstructive or communicating, the former being treated with endoscopic third ventriculostomy (ETV) and the latter by shunt surgery. However, particularly some contradictory observations in paediatric patients have challenged this traditional view. For example, ETV is sometimes successful in communicating hydrocephalus, although the success rate is lower in infants. Furthermore, symptoms of some congenital conditions may present at later stages of life, for example as LOVA or arachnoidal cysts. The concept of communicating *versus *obstructive hydrocephalus has been challenged. This suggests that the variable sites for obstruction to flow may be important for treatment, and that there could be a number of parallel flow routes. Here, the role of sub- or microstructural water transport mechanisms at the molecular level is still unclear: how they relate and add to the known ways of CSF absorption and to our current biomechanical understanding of hydrocephalus. These molecular flow routes could be genetically determined and also age-dependent. In the future, treatment paradigms for paediatric hydrocephalus could be modified and improved when more detailed knowledge of the CSF flow routes is forthcoming.

*Paediatric hydrocephalus: a survey and some thoughts with regard to improve treatment*. Regina Eymann

In recent time, attempts to understand pathophysiology of hydrocephalus developed from a mechanical concept into a concept focusing on compliance. With the help of dynamic infusion tests, pulse wave analysis, pulsatile hydrodynamics we try to get another understanding of the pathophysiology of hydrocephalus. But the disease pattern, especially in children, is not entirely understood. Nevertheless we implant shunts and perform external ventricular drains (ETV). There are three major complications in shunt therapy: over drainage, shunt infections and foreign body reaction to the silicone catheters. Problems with complications are:

1. To this day there is no clear definition of the phenomenon over drainage.

2. Shunt infection is not clearly defined, and there is no consensus about the therapy.

3. Foreign body reaction against silicone: it was possible to mimic this in an animal model.

Is the ETV really the solution for these problems or do we provoke longstanding overt ventriculomegaly (LOVA)? Up to now we have no instrument to monitor ICP online telemetrically in individual patients.

## Conclusion

*Conference Summary*. Anthony Marmarou

Almost half of the contributions to Hydrocephalus 2008 reported clinical studies of which 17% were paediatric; the other half mostly consisted of laboratory and technical contributions, showing that hydrocephalus embraces multi- and trans-disciplinary research. The conference also supported translational aspects of hydrocephalus research. Hydrocephalus research has made gains in the field of advanced imaging techniques, genetic and stem cell therapies, CSF biomarkers, A-beta clearance and in disease modelling. However, the search for adequate and more natural experimental models needs to continue. In the clinical setting, it appears that there is a greater appreciation for diagnostic accuracy of idiopathic normal pressure hydrocephalus (iNPH), and the use of supplementary tests has increased in accordance with the guidelines. In general, non-invasive technology has made significant advances and progress, e.g. bold signal imaging, magnetic resonance spectroscopy, and diffusion tensor imaging. Mathematical modelling has progressed in teasing out both compliance and outflow resistance from volume, flow and pulsatility in the ventricles and in the subarachnoid space, in pulsatility theory and in the aqueductal flow. Furthermore, valve design and technical equipment are improving, allowing selection for the individual clinical situation and for more reliable and practical assessment of shunt function. We are approaching a > 90% favourable outcome of shunt treatment with a low frequency of complications. Experience in endoscopic third ventriculostomy (ETV) has increased from the classical indications to secondary and idiopathic NPH.

Unfortunately, the low number of patients in many clinical studies makes it difficult to arrive at sound conclusions and the need for prospective multicenter studies and collaboration among centres and investigators needs to be stressed. Among the 74 clinical papers presented, there were only 2 concerning prospective randomized trials, 5 prospective controlled trials (4 Shunt related) and only 1 potentially eligible for Class 1 trial. Paediatric studies were in the minority and a better balance is needed. The big question is, why, despite accurate diagnosis and state of the art management, there are still patients that do not improve. Are these failures refractory to treatment or just beyond the window of opportunity? The exclusiveness of shunt treatment, e.g. in NPH, needs a placebo trial for the effect of shunts or a prospective randomized double-blind multicenter trial: shunt *versus *ETV in patients eligible for either procedure. As further vignettes of the conference, neuropsychology, cognitive assessment and functional neuroimaging provided promising and relevant measures of the disease, as well as differential diagnosis and assessment of outcome after treatment. Epidemiological risk factors for hydrocephalus do exist; however, race does not appear to be important. Proteomic analysis of CSF can differentiate between iNPH and Alzheimer's disease (AD).

The headline news was that Binswanger's and AD, alone or coexisting with iNPH, do not preclude a shunt. As one example, the *post hoc *analysis of a clinical trial for shunting of early AD patients using a different global measure, the Alzheimer's Disease Cooperative Study-Activities of Daily Living Inventory (ADCS-ADL) showed benefit to the test group. Furthermore, new approaches for CSF drug delivery (*ex-vivo*) and gene therapy are available and have shown protection against neurotoxins in experimental studies. Adjunct neuromodulative or neurorestorative treatment in the human disease is anxiously awaited.

One hopeful sign is that the number of young investigators has increased. Support is needed to create an environment that encourages research and facilitates publication to expand the literature and increase awareness of the disease. This can only be done by collaborative effort among researchers and professionals from various disciplines and allied health professionals, all with a common language. This needs a platform, and as a further outcome of this meeting, the newly created International Society of Hydrocephalus and CSF Disorders (ISHCSF) was inaugurated. As such, task forces are required as soon as possible for Nomenclature, Classification, Consensus on Outcome Scales and Quality of Life measures, Standardized Disease Assessment, Imaging and Reporting of Complications. Bearing in mind that hydrocephalus is one of the more complex injuries to the most complex organ, why should we expect a simple solution? We simply have to keep trying.

## Competing interests

The authors declare that they have no competing interests.

## Authors' contributions

The authors contributed equally to this meeting report. Both authors have read and approved the final version of this manuscript.

## Supplementary Material

Additional file 1 Invited Speakers and Affiliations. Click here for file
