# Supplementary material for: Hydrocephalus 2008, 17–20th September, Hannover Germany: a conference report
Source: Cerebrospinal Fluid Res. 2008 Dec 16;5:19. doi: 10.1186/1743-8454-5-19 (PMC2615740; doi:10.1186/1743-8454-5-19)
Supplement: Additional file 1 — Invited Speakers and Affiliations. [file 1743-8454-5-19-S1.doc]

## Invited speakers and affiliations

#### Andersson, Nina, Dept of Biomedical Engineering and Informatics, Umeå Univ Hospital, 901 85 Umeå, Sweden

#### Bayston, Roger, Div Orthopaedic and Accident Surgery, Queen's Medical Centre, Nottingham Univ., Nottingham, NG7 2UH, UK

Black, Peter M, Dept Neurosurgery, Brigham and Women´s Hospital, Boston, MA 02115 USA

Brinker, Thomas, Dept Neurosurgery, Int. Neuroscience Institute Hannover, 30625 Hannover, Germany

#### Cesarini, Kristina, Dept Neurosurgery, Univ Hospital Uppsala, 751 85 Uppsala, Sweden

#### Czosnyka, Zofia, Dept Clinical Neurosciences, Addenbrooke´s Hospital, CB2 2QQ Cambridge, UK

#### D’Avella, Domenico, Dept Neurosurgery and Neuroscience, Univ of Padova, 35128 Padova, Italy

#### De Deyn, Peter Paul, Dept Neurology, General Hospital of Neurology, B-2020 Antwerp, Belgium

Del Bigio, Marc, Dept Pathology (Neuropathology), University of Manitoba, Winnipeg, R3E 3P5 Manitoba, Canada

#### Eklund, Anders, Dept Biomedical Engineering and Informatics, Umeå Univ Hospital, 901 85 Umeå, Sweden

#### Eymann, Regina, Dept Neurosurgery, Saarland Univ, Medical School, 66421 Homburg/Saar, Germany

#### Gangemi, Michelangelo, Dept Neurological Sciences, Section of Neurosurgery, Federico II Univ School of Medicine, 80131 Naples, Italy

#### Hellström, Per, Inst Neuroscience and Physiology, Sahlgrenska Univ Hospital, 413 45 Göteborg, Sweden

Johanson, Conrad E, Dept Neurosurgery, Rhode Island Hospital, Providence, RI 02903 USA.

Juhler, Marianne, Dept Neurosurgery, Rigshospitalet, 2100 Copenhagen, Denmark

#### Kiefer, Michael, Dept Neurosurgery, Saarland Univ, Medical School, 66421 Homburg-Saar, Germany

#### Kranz, Dory, Hydrocephalus Association, 870, Market St, # 705, San Francisco, CA 94102, USA

Krauss, Joachim K, Neurosurgical Dept, Hannover Medical School, 30625 Hannover, Germany Luciano, Mark, Dept Neurosurgery, Cleveland Clinic Foundation, Cleveland, OH 44195 USA

Malm, Jan, Dept Clinical Neuroscience, Umeå Univ Hospital, 901 85 Umeå, Sweden

Marmarou, Anthony, Dept Neurosurgery, Virginia Commonwealth Univ Medical Center, Richmond, VA 23298-0508 USA

McAllister II, James Pat, Dept Neurosurgery, Div Pediatric Neurosurgery, Univ of Utah, Salt Lake City, UT 84132-2303 USA

#### Münte, Thomas, Dept Cognitive Neurology and Neurophysiology, Medical School, Otto-von- Guericke Univ 39106 Magdeburg, Germany

#### Oi, Shizuo, Dept Neurosurgery, Jikei Univ School of Medicine (JWCMC), 105-8461 Tokyo, Japan

Pickard, John D, Neurosurgery Unit and Dept Clinical Neurosciences, Addenbrooke´s Hospital, CB2 2QQ Cambridge, UK

Rekate, Harold L, Div Neurosurgery ,Barrow Neurological Institute, St. Joseph's Hospital and Medical Center, Phoenix, AZ 85013 USA

#### Relkin, Norman, Dept Neurology and Neuroscience, Weill Medical College, New York Presbyterian Hospital, New York, NY 10021 USA

Richards, Hugh K, Academic Neurosurgery Unit, Univ Cambridge, Addenbrooke´s Hospital, Cambridge CB2 2 QQ, UK

#### Romner, Bertil, Dept Neurosurgery, Copenhagen Univ Hospital, Rigshospitalet, 2100 Copenhagen, Denmark

#### Sahuquillo, Juan, Div Neurosurgery, Neurotraumatology and Neurosurgery Res Unit, Vall d’Hebron Univ Hospital, 08023 Barcelona, Spain

Samii, Madjid, Dept Neurosurgery, Int Neuroscience Institute Hannover, Rudolf-Pichlmayr-Str.4, 30625 Hannover, Germany

Schuhmann, Martin U, Dept Neurosurgery, Eberhard Karls Univ of Tübingen, Medical School

72076 Tübingen, Germany

Silverberg, Gerald D, Dept Neurosurgery, Stanford Univ Medical Center, Stanford, CA 94305 USA

#### Stolze, Henning, Dept Neurology, Ev-Luth Diakonissenanstalt zu Flensburg , 24939 Flensburg, Germany

#### Stopa, Edward, Depts Pathology & Clinical Neuroscience, Warren Alpert Medical School of Brown University, Providence, RI 02903 USA

Tisell, Magnus, Inst Neuroscience and Physiology, Sahlgrenska Academy, Univ of Gothenberg, 413 45 Göteborg, Sweden

#### Tullberg, Mats, Inst Neuroscience and Physiology, Sahlgrenska Academy, Univ of Gothenberg, 413 45 Göteborg, Sweden

#### Wikkelsö, Carsten, Inst Neuroscience & Physiology, Sahlgrenska Academy, Univ of Gothenberg, 413 45 Göteborg, Sweden

Williams, Michael A, LifeBridge Health Brain & Spine Inst, Sinai Hospital, Baltimore, MD 21209 USA
